# Endometriosis classification, staging and reporting systems: a review on the road to a universally accepted endometriosis classification †,‡

**DOI:** 10.52054/FVVO.13.3.025

**Published:** 2021-12-30

**Authors:** International Working Group of AAGL, ESGE, ESHRE and WES, N Vermeulen, M.S. Abrao, J.I. Einarsson, A.W. Horne, N.P. Johnson, T.T.M. Lee, S Missmer, J Petrozza, C Tomassetti, K.T. ZonderVan, G Grimbizis, R.L. de Wilde

**Affiliations:** ESHRE, Central office, Meerstraat 60, Grimbergen, BE 1852, Belgium; Disciplina de Ginecologia, Departamento de Obstetricia e Ginecologia, Faculdade de Medicina FMUSP, Universidade de Sao Paulo, , São Paulo, Brazil; Gynecologic Division, BP - A Beneficencia Portuguesa de Sao Paulo, São Paulo, SP, Brazil; Brigham and Women’s Hospital, Department of Obstetrics and Gynecology, Division of Minimally Invasive Gynecologic Surgery, Boston, MA, USA; University of Edinburgh, MRC Centre for Reproductive Health, QMRI, 49 Little France Crescent, Edinburgh, UK EH16 4TJ; Robinson Research Institute, University of Adelaide, Adelaide, South Australia; Magee Womens Hospital of UPMC, Department of Obstetrics, Gynecology and Reproductive Sciences, Pittsburgh, PA, USA; Michigan State University College of Human Medicine, Department of Obstetrics, Gynecology and Reproductive Biology, East Lansing, MI, USA; Harvard University T H Chan School of Public Health, Department of Epidemiology, Boston, MA, USA; World Endometriosis Research Foundation, WERF, London, UK; Massachusetts General Hospital Fertility Center, Department of Obstetrics and Gynecology, Boston, MA, USA; University Hospital Leuven, Department of Obstetrics and Gynaecology, Leuven University Fertility Centre, Leuven, Belgium; University of Oxford, Oxford Endometriosis CaRe Centre, Nuffield Department of Women’s & Reproductive Health, Oxford, Oxfordshire, UK; University of Oxford, Wellcome Centre for Human Genetics, Oxford, Oxfordshire, UK; Medical School, Aristotle University of Thessaloniki, 1st Dept Obstet Gynecol, Tsimiski 51 Street, Thessaloniki, Greece 54623; Carl von Ossietzky Universitat Oldenburg, University Hospital for Gynecology, Oldenburg, Germany.

## Abstract

**Background:**

In the field of endometriosis, several classification, staging and reporting systems have been developed. However, endometriosis classification, staging and reporting systems that have been published and validated for use in clinical practice have not been systematically reviewed up to now.

**Objectives:**

The aim of the current review is to provide a historical overview of these different systems based on an assessment of published studies.

**Materials and Methods:**

A systematic Pubmed literature search was performed. Data were extracted and summarised.

**Results:**

Twenty-two endometriosis classification, staging and reporting systems have been published between 1973 and 2021, each developed for specific and different purposes. There is still no international agreement on how to describe the disease. Studies evaluating different systems are summarised showing a discrepancy between the intended and the evaluated purpose, and a general lack of validation data confirming a correlation with pain symptoms or quality of life for any of the current systems. A few studies confirm the value of the Enzian system for surgical description of deep endometriosis. With regards to infertility, the endometriosis fertility index has been confirmed valid for its intended purpose.

**Conclusions:**

Of the 22 endometriosis classification, staging and reporting systems identified in this historical overview, only a few have been evaluated, in 46 studies, for the purpose for which they were developed. It can be concluded that there is no international agreement on how to describe endometriosis or how to classify it, and that most classification/staging systems show no or very little correlation with patient outcomes.

**What is new?:**

This overview of existing systems is a first step in working towards a universally accepted endometriosis classification.

## Introduction

Endometriosis is an inflammatory oestrogen- dependent disease associated with chronic pelvic pain and/or infertility that is characterised by lesions of endometrial-like tissue outside of the uterus (Johnson et al., 2017). The disease is usually confined to the abdominal cavity but, rarely, extra-abdominal lesions have been detected in the lungs, brain and even in the eye. Within the pelvic cavity, the variety of presentations is extensive with lesions detected on the peritoneum, within the ovaries (endometrioma), around the uterus, but also affecting the urinary tract, bowel, and vagina. Most definitions, but not all, consider adenomyosis (similar lesions arising within the myometrium) as a separate disease (Zegers-Hochschild et al., 2017).

Traditionally, three phenotypes of endometriosis lesions are recognised; peritoneal, ovarian (endometrioma) and deep endometriosis (DE) (Working group of ESGE ESHRE and WES, et al., 2020a, Working group of ESGE ESHRE and WES, et al., 2020b, Working group of ESGE ESHRE and WES, et al., 2017a, Working group of ESGE ESHRE and WES, et al., 2017b). Symptoms include chronic pelvic pain (dysmenorrhea, acyclic pelvic pain, dyspareunia, dyschezia, dysuria) with severity ranging from mild to debilitating, infertility, and non-specific symptoms (fatigue), but endometriosis can also be asymptomatic (Zondervan et al., 2020). Treatment options for pain include different medical and hormonal treatments or surgery, while for infertility, surgery and/or ART have been used.

Since the first descriptions of endometriosis, this spectrum of lesions and symptoms has urged clinicians to attempt to classify the disease into informative subgroups or hierarchical stages. By definition, classification entails a systematic arrangement of similar entities on the basis of certain differing characteristics (Miller-Keane and O’Toole, 2005). When disease classification can be related to treatment outcomes or prognosis, the system is considered a staging system.

In the field of endometriosis, several classification, staging and reporting systems have been developed. The current paper provides, based on an assessment of published studies, a historical overview of these different systems. Validation studies and published reports on the implementation of the different classification, staging and reporting systems have been summarised to highlight the uptake, benefits and drawbacks of published systems for endometriosis.

## Materials and Methods

**Figure 1:**
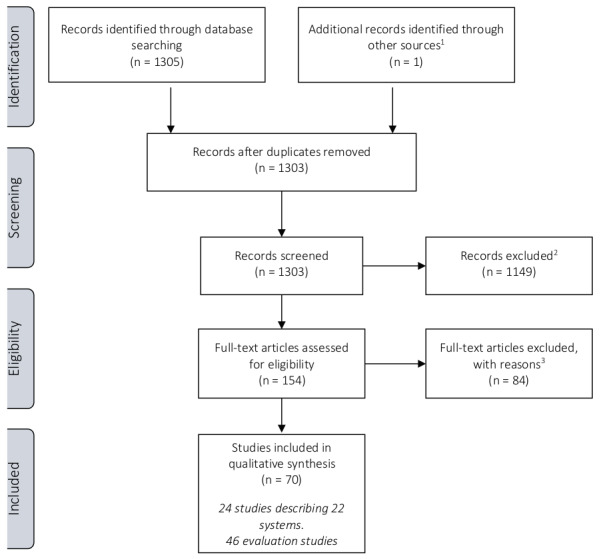
— PRISMA flow diagram for the selection of studies describing endometriosis classification, staging and reporting systems. 1 The recent paper on #ENZIAN was included, although published after the inclusion deadline. 2 Non-English language studies, animal studies and papers not focusing on endometriosis. 3 Exclusion criteria included: Full text not able to be retrieved (n=9); Publication types [case report, expert opinion, editorial] (n=28); Relevant patients are not included [not endometriosis] (n=2); Relevant intervention/outcomes are not assessed [not on classification] (n=35); Irrelevant (n=6); Not English (n=4).

A literature review was performed collecting studies and reports focusing on “endometriosis” and “classification, staging, or scoring”. PUBMED/ MEDLINE was searched, and studies were included from inception (1966) up to 08/05/2020; all retrieved references were checked for relevance. Non-English language studies, animal studies and papers not focusing on endometriosis, including those focusing specifically on adenomyosis, were excluded from the retrieved references. Papers and classifications systems focusing on endometriosis but including adenomyosis were not excluded. For the remaining references, the full text papers were collected and assessed. Inclusion criteria included original studies focusing on endometriosis and classification, staging or reporting systems. The results of the literature search are summarised in a PRISMA flowchart (Figure 1). The details of the final set of papers are summarised in evidence tables. The draft paper was published for stakeholder review by all societies involved; 81 comments were tabulated in a review report and, where relevant, incorporated in the final version of the paper.

## Results

The literature review retrieved 1305 references;one reference was added at a later stage. After applying the exclusion criteria, 154 full paperswere assessed, of which 84 papers were excluded for the following reasons: full text papers could notbe retrieved (n=9), not written in English (n=4), inappropriate publication types (case report, expertopinion, editorial) (n=28), and relevant patients and/or intervention/outcomes are not assessed(not endometriosis or not classification) (n=43).Seventy papers were included for either describing a classification, staging or reporting system inendometriosis (n= 24) or evaluating one (n=46)(Fig. 1). The systems in endometriosis described in this paper have been published as classification, staging or reporting systems, even though somewere developed for stratification or subgrouping rather than classification.

Table I provides an overview of the 22 classification, staging or reporting systems identified in the literature and included in this report. The 46 studies reporting an evaluation of the different systems are listed in Table II.

**Table I:** Historical overview of endometriosis classification/staging system.

Endometriosis classification/staging system	Publication year	Classification/staging based on	Intended purpose							Details	Reference
			Diagnosis/Pre-op assessment	Description	Staging	Treatment selection	Prediction of difficulty of surgery	Prediction of pain remediation/improved QoL	Prediction of conception	
#ENZIAN classification	2021	Surgical observation or imaging	√	√						Non-invasive and surgical description system for endometriosis	(Keckstein et al., 2021)
Adhesion scoring system	2020	US	√						√	Pre-operative prediction of the pelvic adhesion status	(Ichikawa et al., 2020)
ENDOGRAM	2019	Disease markers in biopsy sample				√				Analysis of endometriotic tissues supporting therapeutic decisions	(Bouquet de Joliniere et al., 2019)
ENDORECT	2019	Clinical examination, US, MRI	√							Preoperative score to predict rectosigmoid involvement	(Chattot et al., 2019)
Bowel Endometriosis Syndrome (BENS) score	2017	Symptoms	√							Identify bowel endometriosis syndrome based on patient reported symptoms and QoL	(Riiskjaer et al., 2017)
Preoperative ultrasound-based endometriosis staging system (UBESS)	2016	US	√				√			Pre-operative staging and prediction of the level of complexity of laparoscopic surgery.	(Menakaya et al., 2016)
Classification of ureteral endometriosis	2015	Surgical observation	√	√	√					Clinical classification of urinary tract endometriosis	(Knabben et al., 2015)
EPHect SSF - EPHect MSF (surgical form)	2014	Surgical observation		√						Recording of surgical phenotypic information and related sample collections obtained at laparoscopy	(Becker et al., 2014)
Clinical score	2014	Symptoms	√				√			Predict DE presence before endometrioma surgery	(Lafay Pillet, et al., 2014)
LSD/MURO Classification	2013	Modified Virtual Colonoscopy	√							Descriptive imaging classification, with implied severity for rectogenital and disseminated endometriosis	(van der Wat et al., 2013)
ECO system	2012	Extent, symptoms and objectives				√				Determine most appropriate management	(Lasmar et al., 2012;2015)
Deep endometriosis staging form	2011	US			√					Staging system for DE based on ultrasonographic finding	(Coccia and Rizzello, 2011)
Endometriosis Fertility Index (EFI)	2010	Surgical observation + Patient parameters							√	Prediction of (non-IVF) pregnancy after surgery	(Adamson and Pasta, 2010)
ENZIAN classification	2005	Surgical observation (or MRI)	√^1^	√						Surgical classification for DE	(Keckstein et al., 2021, Tuttlies et al., 2005)
Chapron classification	2003	Surgical observation		√			√			Surgical classification for DE with suggested operative procedure	(Chapron et al., 2003a;2003b)
Revised ASRM classification	1997	Surgical observation		√	√					Adapted from rAFS score	(American Society for Reproductive Medicine, 1997)
TOP classification	1993	Surgical observation			√				√	Evaluate the severity of endometriosis by site, i.e., fallopian tubes (T), ovaries (O) and the peritoneum (P) and impact on PR	(Kurata et al., 1993)
Revised American Fertility Society (AFS) classification	1985	Surgical observation		√						Point system to determine stage/degree of endometriosis involvement	(American Fertility Society, 1985)
American Fertility Society (AFS) classification	1979	Surgical observation		√							(American Fertility Society, 1979)
Buttram classification	1978	Surgical observation		√					√	Classification of endometriosis in the infertile female (expanded from (Acosta, et al., 1973))	(Buttram, 1978)
Kistner classification	1977	Surgical observation		√					√	Classification as a tool to link pregnancy rates with the presence/extent of disease	(Kistner et al., 1977)
Acosta classification	1973	Surgical observation		√					√	Classify the extent of disease and relationship with pregnancy rate	(Acosta et al., 1973)

DE, deep endometriosis; ECO, disease extent complaints, objectives system, QoL, Quality of life; PR, pregnancy rate; US, ultrasound, #ENZIAN: the recently updated ENZIAN classification, which incorporates all types of endometriosis

^1^In case of ENZIAN score based on MRI.

**Table II:** Historical overview of endometriosis classification/staging systems.

Diagnosis/Pre-op assessment	Description	Staging	Treatment selection	Prediction of difficulty of surgery/complication	Prediction of pain remediation/QoL	Prediction of conception	Feasibility	Interobserver agreement	Aim of the study	Sample size	Age mean (range or SD)	Population source	Endometriosis case definition	Main results	Reference
ENZIAN															
				+ length of hospital stays					Correlation with Clavien-Dindo complication grading	401	34.8 years (SD 8.73)	Single-centre	Histologic confirmation	ENZIAN A2,C1,C3 and FA were risk factors for the length of hospital stay.	(Nicolaus et al., 2020)
+									ENZIAN (MRI) Correlation with intraoperative findings	63	33.5 years (22-49)	2 centres	Surgical confirmation	Sensitivity and NPV of MRI confirmed by surgerey were 95.2% and 91.7% (lesions in the vaginal/rectovaginal space), 78.4% and 56% (utero-sacral ligaments), 91.4% and 89.7% (rectum/sigmoid colon), 57.1% and 94.1% (myometrium), 85.7% and 98.3% (bladder), and 73.3% and 92.2% (intestine), resp.	(Burla et al., 2019)
	No analysis								Application of the rENZIAN system	60	30.5 years (28.6-32.3)	Single-centre	Laparoscopic diagnosis	Medial compartment was found as the most affected one in 80% of the cases (mainly ovarian endometriomas), followed by posterior compartment in 65% and less frequent, anterior compartment.	(Morgan-Ortiz et al., 2018)
+									ENZIAN (MRI) Accuracy of the score compared to surgical-pathological findings	115	36 years (20.3-48)	Single-centre	Histologic confirmation	The sensitivity, specificity, accuracy, PPV and NPV of MRI were 94%, 97%, 95%, 99%, 86%, resp. The highest accuracy was for adenomyosis (100%) and endometriosis of utero-sacral ligaments (98%), slightly lower for vagina-rectovaginal septum and colorectal walls (96%), and the lowest for bladder endometriosis (92%). The concordance with histopathology was excellent.	(Di Paola et al., 2015)
				+ Operating time					Preoperative estimation of laparoscopic operating time	151	31 years (19-53 years)	Single-centre	Histologic confirmation	An ENZIAN-based model for estimating operating time for DE, assuming complication-free procedures (model's predictive power: P<0.001). The error of estimation for the operating time prediction is 0±35.35 min (range-83 to +117min).	(Haas et al., 2013a)
	+								Identification of duplicate classifications of the same lesions	219	Not reported	Single-centre	Histologic confirmation	Comparison to rAFS: The severity of DE according to ENZIAN was as follows: grade 1: 45%; grade 2: 26%; grade 3: 19%; grade 4: 10%. 58 patients were classified according to ENZIAN although they did not fulfil the criteria of DE and had previously been classified according to the rAFS classification. Adaptation of the ENZIAN score would reduce the diagnoses of DE by 36% (95% CI: 29%-44%).	(Haas et al., 2011)
UBESS															
				+					Correlation with RCOG laparoscopic level of complexity 1, 2, and 3	293	Not reported	Multi-centre	history of chronic pelvic pain, or endometriosis.	Strengths: UBESS predicted the requirement for RCOG level 3 laparoscopic surgical skills (accuracy, 89.4%-95.4%). Limitation: misclassification of women who require surgical ureterolysis in the absence of bowel DE	(Espada et al., 2020)
				-					Correlation with the difficulty of the surgery	33	32.8 years (SD 7.7)	Single-centre	Histologic confirmation	Weak concordance between pre-op UBESS score and the difficulty of the surgery (RCOG, concordance Kendall Tau 0.22) and between UBESS and CHI (concordance 0.30).	(Chaabane et al., 2019)
				+ surgical skills					Validation for predicting the correct RANZCOG/AGES' laparoscopic skill level.	155	32.7 years (SD 8.6)	Multi-centre	history of chronic pelvic pain and/or endometriosis	The accuracy, sensitivity, specificity, PPV and NPV, and positive and negative likelihood ratios of the UBESS I to predict the RANZCOG/AGES surgical skill levels 1/2 were 99.4%, 98.9%, 100%, 100%, 98.5%, not applicable, and .011; those of UBESS II to predict surgical skill levels 3/4 were: 98.1%, 96.8%, 98.4%, 93.8%, 99.2%, 60 and .033, and those for UBESS III to predict surgical skill level 6 were: 98.7%, 97.2%, 99.2%, 97.2%, 99.2%, 115.7, and 0.028. The rate of correctly predicting the exact level of skills needed was 98.1%, and Cohen's kappa statistic for the agreement between UBESS prediction and levels of training required at surgery was 0.97, indicating almost perfect agreement.	(Tompsett et al., 2019)
EFI															
						+			Accuracy for the prediction of non-ART pregnancy	4598	NA	NA		Cumulative non-ART pregnancy rate at 36 months increased from 10% (95% CI: 3, 16%) in women with EFI score 0-2 to 69% (95% CI: 58, 79%) in women with EFI score 9-10, with a significant increase for each score category (0-2, 3-4, 5-6, 7-8, 9-10)	(Meta-analysis) (Vesali et al., 2020)
								Acceptable	Reproducibility among 3 experts	82	Reproductive age as inclusion citerium	Single-centre	Surgical confirmation	A near 'inter-expert' clinical agreement rate (1.000, 95% CI 0.956-1.000;P=0.0149) was observed. The numerical agreement between two experts was also high (0.988, 95% CI 0.934-1.000); similarly, high agreement rates were observed for both 'junior-expert' comparisons (clinical 0.963, 95% CI0.897-0.992; numerical 0.988, 95% CI0.934-1.000) and 'intra-expert' comparisons (clinical 0.988, 95% CI 0.934-1.000; numerical 1.000,95% CI0.956-1.000).	(Tomassetti et al., 2020)
						+ Conception rate			Accuracy for the prediction of pregnancy	123	32.4 years (no range)	Single-centre	Surgical confirmation	8 (40%) patients with low, 20 (58.82%) with moderate, and 26 (96.29) with high EFI conceived. EFI score showed statistically significant positive correlation with pregnancy outcome P=0.001. Patients conceived spontaneously, after ovulation induction (+/- IUI) or after IVF	(Negi et al., 2019)
						+			Accuracy for the prediction of non-ART pregnancy in recurrent endometriosis	107	31.1 years (SD 0.39)	Single-centre	Surgical confirmation - recurrent endometriosis	Cumulative pregnancy rates (CPR) during the first 2 years were 51.86% in women with EFI≥5, and 26.00% in women with EFI<5. At 3- and 5-years post-surgery, the CPR increased further in women with EFI ≥ 5, but not in women with EFI < 5. The EFI score had good predictive power for postoperative pregnancy in women with recurrent endometriosis	(Zhou et al., 2019)
						+			Accuracy of the prediction of non-ART pregnancy	68	XX	Single-centre	Surgical confirmation	The mean EFI scores of 68 women who were not pregnant and pregnant were 5.43 +/-0.36 and 6.88 +/-0.28, respectively. The relation between EFI and natural pregnancy was significant (cumulative overall PR, p= 0.006), whereas rAFS stage was not (univariate logistics, p= 0.853). The cut-point for maximum natural pregnancy outcomes was 6(area under ROC curve=0.710, 95% CI 0.586-0.835)	(Kim et al., 2019)*
						+			Accuracy for the prediction of non-ART pregnancy	1097	29.8 years (20-46)	Single-centre	Surgical confirmation	The difference in Cumulative pregnancy incidence among EFI scores 10, 7-9, 4-6, and 2-3 was statistically significant (Kaplan-Meier survival analysis). A significant relationship was found between EFI and time to achieve pregnancy	(Zhang et al., 2018)*
						+			Accuracy for the prediction of non-ART pregnancy	235	34 years (20-47)	2 centres	Histologic confirmation	The EFI was highly associated with live births (P<0.001): for EFI of 0-2, the estimated cumulative non-ART LBR at 5 years was 0% and steadily increased up to 91% with an EFI of 9-10, while the proportion of women who attempted ART and had a live birth, steadily increased from 38 to 71% among the same EFI strata (P=0.1). A low least function score was the most significant predictor of failure, followed by having had a previous resection or incomplete resection, being older than 40 compared to <35 years, and having leiomyomas.	(Maheux-Lacroix et al., 2017)*
						*			Accuracy for the prediction of non-ART pregnancy and ART pregnancy	196	32.3 years (SD 4.8)	Single centre	Surgical confirmation	The cumulative PR was 76%. The PR, non-ART PR and ART PR for EFI ≤4 were 42.3%, 0% and 50%; for EFI 5-6, 67.9%, 30.5% and 60.6%; and for EFI ≥7, 87.7%48.2% and 80.3%, resp. The benefit of ART was inversely correlated with the mean EFI score. On multivariate analysis, the EFI score was significantly associated with non-ART pregnancy (OR 1.629,95% CI 1.235-2.150).	(Boujenah et al., 2017)*
			+ ART outcome			+			Accuracy for the prediction of non-ART pregnancy + use for treatment selection (Surgery vs surgery + IVF-ET)	345	32.2 years (22.0-45.0)	Single centre	Histologic confirmation	Significant differences in spontaneous PRs among different EFI scores were identified (chi2=29.945,P<0.05). The least function score was proved to be the most important factor for EFI. In patients with an EFI score ≥5 after 12 months from surgery, the cumulative PRs of those who received both surgery and IVF-ET were much higher than the spontaneous PRs of those who received surgery alone (chi2=4.16,ns)	(Li et al., 2017)*
			+ ART outcome			+			Accuracy for the prediction of non-ART pregnancy + use for treatment selection (Surgery vs surgery + IVF-ET)	412	32.5 years (SD 4.6)	Single centre	Histologic confirmation	A significant relationship between EFI and spontaneous PR was observed at 12 months (P=.001). The least function score and complete removal of endometriotic lesions and pelvic adhesions were significantly associated with spontaneous pregnancy (P=.006). Cumulative PR at 18 months was 78.8%.ART benefits were higher for patients with poor EFI.	(Boujenah et al., 2015)*
						+ non-ART/ART outcome			Accuracy for the prediction of non-ART pregnancy and ART pregnancy	104	34.5 years (SD 4.5)	Single centre	Surgical confirmation	Differences in time to non-ART pregnancy for the six EFI groups were statistically significant (log-rank, p= 1.4x 10(-4)). The AUC for EFI as ART outcome predictor was 0.75 (95% CI 0.61-0.89,p= 6.2x 10(-3)), while the best cut-point for pregnancy was 5.5.	(Garavaglia et al., 2015)*
						+			Accuracy for the prediction of non-ART pregnancy	161	32.08 years (22-40)	Single centre	Surgical confirmation	Comparison to rAFS: Significant differences in cumulative PRs were observed among EFI scores (EFI score 0-3, 8.3%; EFI score 4-7 41.2%, and EFI score 8-10 60.9%; chi2=16.254,p<0.001). EFI scores, but not rAFS stage, predict PRs in patients with endometriosis-associated infertility.	(Zeng et al., 2014)*
						+ ART outcome			Ability of the EFI score and rAFS classification for predicting IVF outcomes	199	32.0 years (SD 4.2)	Single centre	Histologic confirmation	Comparison to rAFS; The AUC of the EFI score (AUC= 0.641, Standard Error (SE)=0.039, 95% CI=0.564-0.717, cut-off score=6) was significantly larger than that of the r-AFS classification (AUC= 0.445, SE= 0.041, and 95% CI= 0.634-0.526). The antral follicle count, estradiol level on day of hCG, number of oocytes retrieved, number of oocytes fertilised, number of cleaved embryos, implantation rate, CPR, and cumulative pregnancy rate were greater in the ≥6 EFI score group compared to the ≤5 EFI score group. EFI has more predictive power for IVF outcomes than r-AFS.	(Wang et al., 2013)
						+			Accuracy for the prediction of non-ART pregnancy	233	31.3 years (SD 3.9)	Single centre	Surgical confirmation	Highly significant relationship between EFI and the time to non-ART pregnancy (P= 0.0004), with the K-M estimate of cumulative overall PR at 12 months after surgery equal to 45.5%(95% CI 39.47-49.87)-ranging from 16.67%(95% CI 5.01-47.65) for EFI scores 0-3, to 62.55% (95% CI 55.18-69.94) for EFI scores 9-10. For each increase of 1 point in the EFI score, the relative risk of becoming pregnant increased by 31% (95% CI 16-47%; i.e., HR 1.31). The 'least function score' was found to be the most important contributor to the total EFI score.	(Tomassetti et al., 2013)*
ECO system															
			+						Validation	166	34.0 years (SD 7.2)	2 centres	Histologic confirmation	Among patients, 78 (47.0%) were medically treated and 88 (53.0%) underwent therapeutic laparoscopy. All 3 patients scoring 2 had undergone hormonal treatment. Among 51 patients scoring 3, 49 (96.1%) were clinically managed and 2 (3.9%) underwent surgery. Among 52 patients scoring 4, 26 (50%) had undergone medical treatment and 26 (50%) surgical treatment. All 56 patients who scored 5 and the 4 patients who scored 6 underwent surgery.	(Lasmar et al., 2015)
rASRM / rAFS / AFS															
						-			Accuracy for the prediction of non-ART pregnancy	161	32.08 years (22-40)	Single centre	Surgical confirmation	Comparison to EFI: The cumulative PR 36 months after surgery was 46.6% (stage I, 53.6%; stage II, 36.0%; stage III, 51.7%, and stage IV,41.7%; chi2=4.143, p=0.246). In the 1st year, PRs significantly differed between patients with rAFS stage IV and those with stages I-III (chi2=6.024,p=0.014). rAFS stage did not predict PR in patients with endometriosis-associated infertility.	(Zeng et al., 2014)
						- ART			Ability of rAFS (vs EFI) to predict IVF outcomes	199	32.0 years (SD 4.2)	Single centre	Histologic confirmation	Comparison to EFI: The AUC of the EFI score was significantly larger than that of the r-AFS classification (AUC=0.445, SE=0.041, and 95% CI=0.364-0.526).	(Wang et al., 2013)
				+					Correlation with Clavien-Dindo complication grading	401	34.8 years (SD 8.73)	Single centre	Histologic confirmation	rASRM IV was a risk factors for the length of hospital stay. Clavien-Dindo Grade III complications were significantly associated with rASRM stage IV	(Nicolaus et al., 2020)
								Acceptable	Inter-observer agreement	148	32.0 years (SD 6.7)	Single centre	105 women with and 43 women without a postoperative endometriosis	Surgeons and expert reviewers had substantial agreement on diagnosis and staging after viewing digital images (n=148; mean j=0.67, range 0.61-0.69; mean j=0.64, range 0.61-0.69; mean j=0.64, range 0.53-0.78, resp.) and after additionally viewing operative reports (n=148; mean j=0.88, range 0.85-0.89; mean j=0.85, rane 0.84-0.86, resp). Although additionally viewing MRI findings (n=36) did not greatly impact agreement, agreement substantially decreased after viewing histological findings (n=67), with expert reviewers changing their assessment from a positive to a negative diagnosis in up to 20% of cases.	(Schliep et al., 2017)
					+				Prognostic value of individual adhesion scores for recurrence	379	31.8 years (SD 6.7)	Single centre	histologic confirmation	In endometriosis of advanced stage, younger age at the time of surgery, bilateral ovarian cyst at the time of diagnosis, arAFS ovarian adhesion score > 24, and complete cul-de-sac obliteration were independent risk factors of poor outcomes, and arAFS ovarian adhesion score > 24 had the highest risk of recurrence [hazard ratio=2.948 (95% CI: 1.116-7.789), p=0.029].	(Yun et al., 2015)
						ns AFC, FSH			Correlation with the number of follicles, the level of FSH	39	28.7 years (22-34)	Single centre	Surgical confirmation	No statistically significant correlation between the AFC, the level of FSH and the stage of endometriosis was found.	(Posadzka et al., 2014)
						+ ART outcome			Prediction of IVF outcome	40	34.7 years (SD 4.3)	Single centre	Surgical confirmation	Higher cancelation rates, higher total gonadotropin requirements, and lower oocyte yield were found in women with endometriosis Stage III and IV compared with both the Stage I/II and control groups. The fertilization rate was higher in Stage III/IV endometriosis compared to Stage I/II. CPR and LBR were comparable between patients with endometriosis Stage I/II and control group, whereas they were significantly lower in patients with endometriosis Stage III/IV compared to other two groups.	(Pop-Trajkovic et al., 2014)
						- ART outcome			Correlation of rASRM stage with outcome ART treatment	1764 (11)	Not applicable	Not applicable	Not reported	Comparison of women with Stage-III/IV vs Stage-I/II endometriosis: LBR, RR=0.94 (95% CI, 0.80-1.11); CPR, RR=0.90(95% CI,0.82-1.00); miscarriage, RR=0.99(95% CI,0.73-1.36); number of oocytes retrieved, MD=-1.03(95% CI, -1.67 to -0.39). No relevant difference between Stage-III/IV and Stage-I/II in LBR following ART	(meta-analysis)(Barbosa et al., 2014)
								Acceptable (surgeons)	Interrater and intrarater reliability (8 experts)	148	Not reported	Single centre	Not reported	The intrarater reliability for endometriosis diagnosis among the 8 surgeons was substantial: Fleiss kappa=0.69 (95% CI 0.64-0.74). Surgeons agreed on revised ASRM endometriosis staging criteria after experienced assessment in a majority of cases (mean 61%, range 52-75%) with moderate interrater reliability: Fleiss kappa=0.44 (95% CI 0.41-0.47).	(Schliep et al., 2012)
-									Correlation with symptoms	319	Age categories reported	Single centre	Surgical confirmation, histologic confirmation in 72.9%	A correlation between endometriosis stage and severity of symptoms was observed only for dysmenorrhea (chi2=5.14, P=0.02) and non-menstrual pain (chi2=5.63, P=0.018). However, the point estimates of ORs were very close to unity (respectively, 1.33, 95% CI 1.04-1.71, and 1.01, 95% CI 1.00-1.03). The association between endometriosis stage and severity of pelvic symptoms was marginal and inconsistent.	(Vercellini et al., 2007)
					- pain recurrence + relapse	- pregnancy			Predictive value for response to surgical treatment	537	Age categories reported	Single centre	Histologic confirmation	The cumulative probability of pregnancy at 3 years from surgery was 47% (51% at stage I, 45% at stage II, 46% at stage III and 44% at stage IV; chi2=1.50, ns). The cumulative probability of moderate or severe dysmenorrhea recurrence in 425 symptomatic subjects was 24% (32% at stage I, 24% at stage II, 21% at stage III and 19% at stage IV; chi2=6.39, ns). The cumulative probability of disease relapse was 12% (3% at stage I, 11% at stage II, 11% at stage III and 23% at stage IV; chi2=24.95, P=0.0001).	(Vercellini et al., 2006)
+			+		+ pain				Association with type and severity of pain, and with symptoms after laparoscopic surgery	95	Not reported	Single centre	Surgical confirmation	In patients with AFS≥16; preoperative pain scores were significantly higher for dysmenorrhea (p=0.0022) and deep dyspareunia (p<0.0001) but not for non-menstrual pain. After surgery, dysmenorrhea improved in 43% of cases in patients with AFS <16 vs. 66% with AFS≥16(p=0.0037). For deep dyspareunia, improvement was reported by 33% and 67%, resp (ns). Improvement in non-menstrual pain was not significantly different (67% vs. 56%). Cases with advanced disease benefit the most from laparoscopy.	(Milingos et al., 2006)
-					-				Impact of treatment on pain + association pain scores	181	Not reported	Single centre	Histologic confirmation	No correlation was found between the stage of endometriosis according to R-AFS score and the severity of CPP	(Szendei et al., 2005)
							variable		Comparison of laparoscopic and laparotomic scoring	84	Not reported	Single centre	Surgical confirmation	There was considerable variability in laparoscopic vs laparotomic scoring by the same observer, with largest variability in ovarian endometriosis and cul-de-sac obliteration subscores, and least variability for peritoneum endometriosis. The inter-method variation was sufficient to alter the staging in 34.5% of patients, with a difference of 2 stages in 3.6% of patients. In general, there was fair-to-good agreement (kappa coefficient 0.49).	(Lin et al., 1998)
						- ART pregnancy			Impact of severity of endometriosis on the outcome of IVF	61	33.6 years (SD 3.0) and 34.4 years (SD 4.0)	Single centre	Surgical confirmation	Response to COH and the number, maturity and quality of the oocytes was comparable between stages. Fertilization rates for oocytes of patients with stages III/IV were significantly impaired compared to those in stage I/II (P=0.004). The implantation rate, CPR, and miscarriage rate were comparable between stages I/II and stages III/IV.	(Pal et al., 1998)
								variable	Intraobserver and interobserver variability - 5 experts	20	Not reported	Single centre	Not reported	The grand total score varied with an SD of 13.44 when the videotape of a single patient was rated twice by the same observer and varied with an SD of 17.12 when rated by two observers. The greatest variability occurred in endometriosis of the ovary and cul-de-sac obliteration, with less variability for peritoneum endometriosis and for ovarian and tubal adhesions. Comparison of Intraobserver and interobserver scores resulted in a change in endometriosis stage in 38% and 52% of patients, resp.	(Hornstein et al., 1993)
								acceptable	Reproducibility - 2 experts	315	Not reported	Single centre	Not reported	Good to fair agreement scoring endometriosis between the investigator and the blinded reviewer was noted.	(Rock et al., 1995)
							no analysis		Feasibility of AFS and adnexal score	89	Not reported	Single centre	Surgical confirmation	Suggestion to split class IV in class IV and class V (with higher rate of bilateral adnexal disease/adhesions)	(Canis et al., 1992)
- age, symptoms									Relation with endometriosis-associated symptoms and patients' age.	206	30 years (18-44)	Single centre	Surgical confirmation	No significant differences were found in total endometriosis scores, in active scores or in adhesion scores in different age groups. There was no significant difference in prevalence rate of symptoms for different aspects of endometriosis (implants, cysts or adhesions). AFS score does not reflect the intensity of symptoms.	(Marana et al., 1991)
							variable		Feasibility of measuring endometrioma	52	29.5 years (24-39)	Single centre	na	Cyst diameter was calculated using the geometric formula radius=3 square root of 3V/4 pi where V = volume of liquid aspirated. 8 patients with apparently normal pelvis had endometriosis, and 14 with apparent minimal or mild endometriotic lesions were restaged. Laparoscopic ovarian puncture of enlarged ovaries was important for correct diagnosis and staging of endometriosis.	(Candiani et al., 1990)
						- pregnancy			Relation with pregnancy after therapy	214	Not reported	Single centre	Surgical confirmation	The AFS scale poorly specifies the relation between severity of disease and pregnancy outcome after therapy. A nonparametric monotonic estimator, generating a relationship between AFS score and pregnancy following treatment is shown to improve the discriminatory power of the AFS scale.	(Guzick et al., 1982)
						+ pregnancy			(+ Kistner, Buttram) Prediction of pregnancy	214	28.6 years (17-37)	Single centre	Surgical confirmation	The AFS score revealed significant differences in pregnancy rate only if categories were combined (mild plus moderate versus severe plus extensive, P≤0.05). The AFS system revealed that pregnancy success was significantly reduced if an ovarian endometrioma was greater than 3 cm or had ruptured (P≤0.01).	(Rock et al., 1981)

The symbols should be interpreted as follows; + indicates a significant positive result in a correlation (or similar) test, - indicates a significant negative result in a correlation (or similar) test, ns indicates a non-conclusive/non-significant result in a correlation (or similar) test. The highlighted columns represent the intended purpose of the classification/staging system (as in Table I).

AFS, American Fertility Society, AFC, antral follicle count; AGES, Australasian Gynaecological Endoscopy and Surgery; COH, controlled ovarian hyperstimulation; CPP, chronic pelvic pain; CPR, clinical pregnancy rate; DE, deep endometriosis; EFI, endometriosis fertility index; HR, hazard ratio; IVF-ET, IVF embryo transfer; LBR, live birth rate; K-M, Kaplan Meier; NPV, negative predictive value; PPV, positive predictive value; RANZCOG, Royal Australian and New Zealand College of Obstetricians and Gynaecologists; RR, relative risk; .

* Study included in meta-analysis (Vesali et al, 2020).

### Classification and staging systems

In the 1970s, the first “classification” system for endometriosis originated from a study attempting to describe the results of conservative surgical treatment of endometriosis and hereby classify the extent of the disease and its relationship with the pregnancy rate (Acosta et al., 1973). Later, this classification system was further expanded and submitted for consideration to the American Fertility Society (AFS) (Buttram, 1978). Similarly, a system published by Kistner and colleagues was submitted for endorsement by AFS and the International Federation of Fertility Societies (IFFS) (Kistner et al., 1977). In 1979, AFS published a classification system on behalf of a group of experts including the leading authors of the previous systems (American Fertility Society, 1979). The AFS classification for endometriosis, and later published revised AFS (rAFS) and revised American Society for Reproductive Medicine (rASRM) classification, have been the main standard for classifying endometriosis ever since (American Fertility Society, 1979, American Fertility Society, 1985, American Society for Reproductive Medicine, 1997). The different versions of the AFS/ASRM classification system reflect the progress made in the knowledge on endometriosis.

Later attempts of surgical disease description or staging have focused on disease location - such as urinary tract endometriosis (Knabben, et al., 2015) - or subtypes of the disease - such as DE (Chapron et al., 2003a, Coccia and Rizzello, 2011, Tuttlies et al., 2005): the latter group includes the ENZIAN-Score for classifying DE (Tuttlies, et al., 2005). The recently updated #ENZIAN classification extends the previous ENZIAN score to incorporate all types of endometriosis (Keckstein et al., 2021). The EPHect standard recommended (SSF) and minimum required (MSF) were developed for recording of surgical phenotypic information on endometriosis (Becker et al., 2014)

While these classification systems mainly focused on describing the extent of disease during surgery, some attempted to link these observations to outcomes, such as pregnancy rates, after surgery (American Fertility Society, 1979, American Fertility Society, 1985, American Society for Reproductive Medicine, 1997, Kurata et al., 1993), or indicators for disease management (Chapron et al., 2003a). Another group of classification systems focused on pre-operative assessment of the extent of the disease (Chattot et al., 2019, Ichikawa et al., 2020, Knabben et al., 2015, Lafay Pillet et al., 2014, Menakaya et al., 2016, Riiskjaer et al., 2017, van der Wat et al., 2013), based on either patient-reported symptoms or pre-operative imaging, or a combination of both. The ultrasound-based endometriosis staging system (UBESS) additionally aimed at predicting the complexity of endometriosis surgery (Menakaya et al., 2016), as does the adhesion scoring system in case of pelvic adhesions (Ichikawa et al., 2020).

Two systems aimed specifically at outcome prediction for endometriosis: the ‘disease extent, complaints, objectives (ECO)-system’, aiming to select the most appropriate management based on reported symptoms (Lasmar et al., 2012, Lasmar et al., 2015); and the endometriosis fertility index (EFI), aiming to predict the probability of natural conception after surgery (Adamson and Pasta, 2010). Finally, a recently published study “Endogram” sets out to ‘profile’ endometriosis heterogeneity, based on the assessment of several disease markers in a biopsy sample, with the ultimate aim of guiding therapeutic options (Bouquet de Joliniere et al., 2019).

### Replication, validation, and clinical value of published systems

We retrieved 46 studies, mostly observational, reporting an evaluation of the different classification, staging or reporting systems (Table II). The aims and outcomes of the different studies varied significantly.

Of the included studies, eight reported on the practical aspects of the classification systems, being either the feasibility, or the inter-observer and intra- observer variability. Of these, seven studies focused on the rASRM classification system (Candiani et al., 1990, Canis et al., 1992, Hornstein et al., 1993, Lin et al., 1998, Rock, 1995, Schliep et al., 2017, Schliep et al., 2012), while the most recent one evaluated the reproducibility of the EFI (Tomassetti et al., 2020). Early studies (1990s) reported significant variability in rAFS classification by five independent experts reviewing surgery recordings, specifically with regards to endometriosis of the ovary and cul-de-sac obliteration (Hornstein et al., 1993), although another study from the same period reported good to fair agreement in scoring endometriosis between two experts using photographs or recordings (Rock, 1995). In more recent studies, the rASRM classification system was found to have acceptable inter-observer agreement and inter-rater reliability among surgeons and experts reviewing surgical photographs and/or recordings (Schliep et al., 2017, Schliep et al., 2012). Studies have also focused on the feasibility of specific aspects of the AFS/rAFS/rASRM classification, specifically classifying bilateral adnexal disease (Canis et al., 1992), measuring cyst diameter (Candiani et al., 1990), or the reliability of laparoscopic versus laparotomic scoring (Lin et al., 1998). For the EFI, a near perfect clinical agreement rate between two independent experts (1.000, 95% CI 0.956-1.000) and high agreement between two assessments by the same expert (0.988, 95% CI 0.934-1.000) has been reported (Tomassetti et al., 2020).

The remaining studies (n=37) applied the classification or staging systems to a cohort of patients, evaluating whether the system was reliable with regards to its proposed aim, or evaluating whether the classification could be used for other purposes. The latter was mainly the case for the AFS/rAFS/rASRM classification system, which was developed for surgical staging, but has been evaluated for predicting symptom relief and recurrence after surgery (Milingos et al., 2006, Vercellini, et al., 2006), complications after surgery (Nicolaus et al., 2020), ovarian reserve (Posadzka et al., 2014), time to non-ART pregnancy (Yun et al., 2015), pregnancy outcomes (Guzick et al., 1982, Rock et al., 1981), and the outcomes of ART treatment (Barbosa et al., 2014, Pal et al., 1998, Pop-Trajkovic, et al., 2014). Furthermore, correlation of the AFS/rAFS/rASRM classification system with symptoms before surgery was evaluated (Marana et al., 1991, Szendei et al., 2005, Vercellini et al., 2007). To our knowledge, there are no studies specifically evaluating the feasibility or reliability of the AFS/rAFS/rASRM classification system for its proposed aim, being a descriptive system of surgical documentation of disease.

The EFI, a 10-point scoring system grouped into five categories of risk, has been assessed in 12 studies and one review. It has been mainly assessed for its intended purpose, being prediction of the probability of natural conception after surgery (Boujenah et al., 2015, Boujenah et al., 2017, Garavaglia et al., 2015, Kim et al., 2019, Li et al., 2017, Maheux-Lacroix et al., 2017, Negi et al., 2019, Tomassetti et al., 2013, Wang et al., 2013, Zeng et al., 2014, Zhang et al., 2018, Zhou et al., 2019). Interestingly, in some of these studies an evaluation of the prognostic value of the different factors included in the EFI score was also performed. A meta-analysis summarised these validation studies and evaluated the performance of the EFI score for predicting non-ART pregnancy after endometriosis surgery, observing good predictive value with a pooled estimate for AUC of 0.71 (95% CI 0.65-0.80) (Vesali et al., 2020). Some authors have (additionally) evaluated whether its purpose can be extended to guide patient management, by using it to select patients that would benefit from ART treatments (Boujenah et al., 2015, Li, et al., 2017), and/or predicting the chances of pregnancy from ART treatments (Garavaglia et al., 2015, Wang et al., 2013).

The ECO system has been validated for prediction of management (surgery or medical treatment) in a single study, by the same authors that developed the tool (Lasmar et al., 2015).

The UBESS system, developed for pre-operative staging and prediction of the complexity of surgery, was evaluated in three studies reporting on the latter purpose, i.e. difficulty of surgery (Chaabane et al., 2019, Espada et al., 2020) and prediction of surgical skill levels (Tompsett et al., 2019).

Finally, the ENZIAN classification system, developed as a descriptive system for surgical staging of DE, was evaluated for its purpose in two studies (Haas et al., 2011, Morgan-Ortiz et al., 2018). Another evaluation reported on the correlation between the ENZIAN classification and complications after surgery, classified according to the Clavien-Dindo complication grading (Nicolaus et al., 2020). The use of the ENZIAN classification system was further extrapolated for its use in pre-operative assessment with imaging. Two studies evaluated this MRI- based ENZIAN system (Burla et al., 2019, Di Paola et al., 2015), and a third study reported on a model to predict operation time based on the MRI-based ENZIAN classification (Haas et al., 2013a).

In general, published classification or staging systems have been developed with various intended purposes, ranging from diagnosis (including symptoms) and pre- operative assessment, surgical description or staging, to prediction of surgical difficulty and treatment outcomes (both for pain and infertility). The studies summarised above confirm the surgical value of the ENZIAN system for description and pre-operative assessment of DE, and of UBESS for predicting laparoscopic difficulty. However, most classification/staging systems show no or very little correlation with patient outcomes. The exception is the EFI, which has been consistently shown to provide good predictive value for natural conception after endometriosis surgery. It is notable that the development of the EFI was data driven, whereas the development of most other classification/staging systems was based on expert opinion.

## Discussion

The current paper provides an overview of currently available and published classification, staging and/or reporting systems for endometriosis. We include 22 systems published between 1973 and 2021. Each of the systems was developed for a specific and different purpose. The first systems tried to classify the various forms of endometriosis that were encountered (at the time), and this remains the purpose of more recent systems as there still is no international agreement on how to describe the disease. Next, we summarise published studies evaluating the different classification, staging or reporting systems. From this, we show a discrepancy between the intended and the evaluated purpose, and a general lack of validation data confirming correlation with pain symptoms or quality of life for any of the current endometriosis classification systems. With regards to infertility, the EFI has been confirmed valid for its intended purpose of predicting the probability of natural conception after surgery.

Classification and staging systems are widely used in medicine and have been shown to be valuable in guiding clinical management. Examples include the American Joint Committee on Cancer (AJCC) tumor-node-metastasis (TNM) staging systems for cancer, the Gleason score for prostate cancer, the Braak Staging for Parkinson’s disease, and the ACR/EULAR Classification Criteria for Rheumatoid Arthritis. The ACR/EULAR Classification Criteria for Rheumatoid Arthritis were developed based on data analysis of 3115 patients followed by a consensus process in which determinants for risk of rheumatoid arthritis were selected and grouped into a classification system, which was further refined, and the feasibility was optimised (Aletaha et al., 2010). A review published 2 years afterwards identified 17 articles (total 6816 patients) and 17 meeting abstracts (total 4004 patients) investigating the classification criteria. Only a minority of the articles aimed to validate the system in the intended population, while the other studies extended the target population, used different reference standards or adapted the criteria in the system (Radner et al., 2014). The review findings are similar to the findings of the current review, although in a different field of medicine. The TNM staging system for cancer was developed in the early 1950s, aiming to guide clinical classification of cancer cases by anatomical extent. The philosophy and technique of TNM staging were developed by Professor Denoix and later adopted by international societies (Denoix 1952, Sellers, 1971). The system is currently at its eighth edition (Edge et al., 2010). The system is revised in a 6 or 8-year cycle and changes are implemented based on high- level evidence collected through large datasets. Specifications are available for different types of cancer, and the system has been complemented with a summary staging or classification linked to prognosis and used for treatment planning. In the TNM system for lung cancer, as an example, TNM staging adaptations included the removal of rare findings from the system, and corrections in stage grouping based on survival outcomes (Lim et al., 2018). In addition, the TNM system has been increasingly complemented by molecular marker data that more accurately stratify risk in patients and guide appropriate treatment options. The longevity and update systems applied for the TNM staging, and the value of additional molecular subtype identification, are likely to be important guides for the design of future endometriosis classification and staging systems that correlate with relevant patient outcomes.

Specifically, for endometriosis, previous reviews have summarised and commented on existing classification systems, mainly rASRM, ENZIAN and EFI. It has previously been concluded that the rASRM system has poor correlation with pain, fertility outcomes or prognosis, and that the ENZIAN system has poor correlation with symptoms and infertility (Andres et al., 2018, Haas et al., 2013b, Johnson et al., 2017). The EFI system needs further evaluation with regards to the importance of the different parameters and whether to include the completeness of surgical treatment (Maheux-Lacroix, et al., 2017). The conclusion of previous reviews of classification systems and our overview is consistently phrased as a need for a generally accepted classification with a clear goal/ purpose (Adamson, 2011, Andres et al., 2018, Haas et al., 2013b, Johnson et al., 2017, Rolla, 2019). Yet, as presented in this paper, the goal and purpose of published classification, staging or reporting systems for endometriosis is often ignored when evaluating classification or staging systems, limiting the value of the evaluation studies and of the systems in general.

To our knowledge, this is the first report comparing the outcomes assessed in the studies with the intended purposes of the classification systems. Indeed, we show that the rASRM system has been widely evaluated, often with negative conclusions, but we found no studies evaluating the system for its intended goal, which is descriptive surgical staging. ENZIAN and EFI have been evaluated for their intended purpose, but studies have also evaluated whether they can be applied more widely and for other outcomes. Apart from these three systems, only two other classification systems (UBESS and ECO) have been evaluated for their intended purpose, with no evaluations of the remaining 17 classification systems, preventing them from further dissemination and uptake.

The current review provides an overview of published classification systems and studies evaluating them, but no detailed assessment of all positive and negative aspects of the classification systems, so as not to repeat previous reviews (Johnson, et al., 2017). In addition, we have restricted our overview to classification systems published in peer-reviewed papers and available through PUBMED/MEDLINE. Although locally used and/or unpublished systems are available and can be valuable, the relevance of including them in the current review was considered low, as they would not be widely applied, nor evaluated by (independent) researchers. For universal use of a classification system, it is pivotal that the system is accessible, validated, reliable and reproducible.

Our report includes a summary of evaluation studies assessing these aspects in the different classification systems. Even though we retrieved 46 studies, the value of these evaluations is limited. Apart from the EFI score, the current classification systems have not been thoroughly assessed for validity, feasibility and reproducibility. Moreover, a significant proportion of the evaluation studies have examined the classification systems for purposes other than the one for which they were designed and initially evaluated.

Endometriosis is a challenging disease to classify, as it is known to have different phenotypes and presentations (both with regards to the type of lesions and their location), and various symptoms without a clear link to phenotype or presentation. Moreover, the natural progression of the disease is unknown. There is a perceived need for a validated classification or descriptive system for endometriosis that could support further progress in defining subgroups and more importantly guiding the therapeutic options for women with pain and/ or infertility. Such a system would certainly also progress endometriosis research by unifying patient subgroups and facilitating the development of prognostic and predictive tools.

From this overview it can be concluded that several classification, staging and reporting systems have been developed for endometriosis. A universally accepted categorisation of the disease using the experience from the already existing proposals seems to be needed for clinical and research purposes.

## WHAT DOES THIS MEAN FOR PATIENTS?

Since 1973, clinicians have proposed classification systems for endometriosis, and so far 22 different systems have been developed. Some of these systems focus on symptoms, while others have been developed to document the surgical observations, or predict the outcomes after treatment. Ideally, classification systems are evaluated in a research project to confirm it is useful in clinical management. We found that of the 22 classification systems, few have been evaluated for the purpose for which they were developed. From this review, it can be concluded that there is no international agreement on how to describe endometriosis or how to classify it.
